# The Healthy Mind

**Published:** 1921-04-30

**Authors:** 


### THE HEALTHY MIND.

It should be widely understood that the mind is composed of many parts, in the same way as the body, and it is the proper working of these various parts in relationship to each other that constitutes what is termed the "healthy mind." With regard to heredity, many people go daily about the world with the fear of some mental disorder, because they know that there is some instability in their inheritance. It is true that we tend to inherit every attribute of our parents, but, on the other hand, we only *tend* to do so, and if we know what are our tendencies, then it is our duty to protect ourselves from risks. However unstable a person's inheritance is, if he understands his nervous system he need, in the vast majority of cases, have but little fear.

### The Man and the Child.

A great deal has been heard lately of the repressions of childhood being all-important in the nervous troubles of later life, but, as the lecturer* said, he is glad to say he is not one of those who belong to the Freudian school, whose outlook, he believed, is far too narrow. It is no new truth that man is the product of the child, and it cannot be too strongly emphasised that a happy childhood is essential to a well-balanced adult life. Although there is no need to fear heredity, yet there is danger to a child if it is brought up in a home where the parents are unstable and the environment is an unhappy one. It is the psychological atmosphere of the home and the school which is the foundation of success in after-life far more than any scholarship; intelligence should be the aim of education rather than learning.

### One of the Tests.

There are risks in early brilliancy in children—risks which parents, in the gratification of seeing their children standing well with their instructors and school companions, do not always appreciate. Both in the animal and the vegetable kingdom it will be found that rapid development connotes a short life history. By unwise use, or by intensive pressure, the energy which ought to be spread over years may be, and too frequently is, exhausted rapidly at an early period of life.

Orderliness of mind is all-important; the mind dominated by emotion tends to be " sloppy " and unstable. One of the great tests of a healthy mind is its power of adaptation; the problem is at all times a severe one, for it entails the adjusting of an ever-changing self to an ever-changing world. Failure of adaptation is the outward manifestation of mental regression, and may result from many causes, the more common of which are illness and fatigue. Fatigue is common to all, and should be removed by a night's sleep; but it may become more severe, when it will lead to irritability, lack of power concentration, and restlessness. Irritability is usually the earliest of these symptoms, and most persons tend to explain this away by saying that everyone is irritable, but we cannot get away from the fact that irritability is a symptom of mind disorder, and will increase unless steps are taken to control it; and here there is an important point to be remembered, which, if it were more fully appreciated, would result in there being much less irritability in the world, and the point is that irritability is more harmful to the nervous system of the person who is irritable than to other people who are exposed to it.

Apart from medicinal means, the simplest and surest way of relieving the mind from stress, and to ward off irritability, is by relaxing the voluntary muscles, that is, by making them as limp and loose as possible. In attention and mental concentration, the muscles become tense, and the more concentrated we are the more fixed become the muscles. This involves a great strain on the nervous system and if continued for a length of time the nervous system becomes irritable. We lessen this strain by relaxing the muscles, and by doing this it is possible to work for much longer periods of time than we could otherwise do.

### The Physical Side.

There is another factor on the physical side to which attention should be given, and this is that, with failing nutrition, and consequently, falling bodily weight, a greater capacity for work, accompanied by a feeling of wellbeing, may be experienced, but when the fall in weight reaches a certain stage nervous symptoms suddenly develop. Each person has a standard for himself, and in health that standard should vary only within very narrow limits; any noticeable fall should be regarded as a danger-signal. During the war, owing to food restriction, people lost weight, and would write to the newspapers saying how much better they felt in consequence, but collapse came later, and we are now only just beginning to recover from the stresses of war, of which loss of weight was not a small one, and even now irritability is much more noticeable than it was before the war.

Minds differ, and it is our duty to understand the minds of each other, but above all, it is our duty to understand ourselves and to keep our minds in health. In health the mind is turned outwards, but when it becomes disordered it becomes introverted. We become preoccupied, and one thought may hold us, and instead of being able to turn from one subject to another as occasion demands, our whole mind becomes fixed in one direction.

Mental hygiene, and all that it stands for, is one of the greatest branches of preventive medicine, for it includes within its purview the right understanding of true education; it treats of character formation; it indicates to each man the vocation for which he is best suited; it enables us to know our limitations and how to protect our minds against the various stresses to which we are exposed; by it alone we can hope to unravel the problems of the unemployable and the criminal classes; and even with our present knowledge it points the way by which the number of insane may be reduced by 50 per cent. Mental hygiene is a subject which has been too long neglected.

* Summary of lecture delivered at the Institute of Hygiene, Wednesday, April 20, 1921, by Sir Maurice Craig, M.A., M.D., F.R.C.P.

